# Prediction of Sweet Corn Seed Germination Based on Hyperspectral Image Technology and Multivariate Data Regression

**DOI:** 10.3390/s20174744

**Published:** 2020-08-22

**Authors:** Huawei Cui, Zhishang Cheng, Peng Li, Aimin Miao

**Affiliations:** 1 College of Agriculture and Biology, Zhongkai University of Agriculture and Engineering, Guangzhou 510225, China; cuihuawei@zhku.edu.cn; 2 College of Automation, Zhongkai University of Agriculture and Engineering, Guangzhou 510225, China; chengzhichang@zhku.edu.cn; 3 Department of Electronic Engineering, School of Information, Yunnan University, Kunming 650091, China; lipeng@ynu.edu.cn

**Keywords:** hyperspectral image, vigor identification, sweet corn seed, germination prediction

## Abstract

Vigor identification in sweet corn seeds is important for seed germination, crop yield, and quality. In this study, hyperspectral image (HSI) technology integrated with germination tests was applied for feature association analysis and germination performance prediction of sweet corn seeds. In this study, 89 sweet corn seeds (73 for training and the other 16 for testing) were studied and hyperspectral imaging at the spectral range of 400–1000 nm was applied as a nondestructive and accurate technique to identify seed vigor. The root length and seedling length which represent the seed vigor were measured, and principal component regression (PCR), partial least squares (PLS), and kernel principal component regression (KPCR) were used to establish the regression relationship between the hyperspectral feature of seeds and the germination results. Specifically, the relevant characteristic band associated with seed vigor based on the highest correlation coefficient (HCC) was constructed for optimal wavelength selection. The hyperspectral data features were selected by genetic algorithm (GA), successive projections algorithm (SPA), and HCC. The results indicated that the hyperspectral data features obtained based on the HCC method have better prediction results on the seedling length and root length than SPA and GA. By comparing the regression results of KPCR, PCR, and PLS, it can be concluded that the hyperspectral method can predict the root length with a correlation coefficient of 0.7805. The prediction results of different feature selection and regression algorithms for the seedling length were up to 0.6074. The results indicated that, based on hyperspectral technology, the prediction of seedling root length was better than that of seed length.

## 1. Introduction

Sweet corn (*Zea mays* L. *saccharata*) is a vegetable crop with high nutritional and edible value as it is rich in sugar, various amino acids, vitamins, minerals, and dietary fiber [1,2,3]. Sweet corn has many varieties and they are favored by consumers all over the world, more so than common corn [1]. It has been reported that the planting area of sweet corn in China has gradually expanded in recent years. In 2018, the planting area of sweet corn in China was more than 3000 square kilometers, accounting for 25% of the world crop [4]. With the increasing requirements of production safety and variety reliability, high-quality seeds are the most important issue in the development of the planting industry. However, the conditions during vegetation (soil moisture, temperature, nutrition, pests, and diseases), harvesting (mechanical damage, maturity), and post-harvest (seed drying and storage), which are difficult to control, may have a great effect on the seed vigor. Changes in these factors may result in irreversible seed damage, growth retardation, and severe yield losses.

The determination of seed vigor is a priority in modern seed science and a prerequisite for high yield. Therefore, it is necessary to know the seed vigor before sowing to ensure higher seed germination rate and economic income. Seed vigor is an important index of comprehensive seed germination rate, seedling emergence rate, seedling growth potential, and plant stress resistance [1,2]. Therefore, the establishment of a fast, nondestructive, and high-precision method for seed vigor detection before sowing is of great biological and economic significance for ensuring seed quality, optimizing crop production facilities, and improving crop yield.

Traditional methods for seed vigor evaluation include immunoassay tests [5], polymerase chain reaction tests [6,7], and germination tests [8,9]. However, the above chemical methods or planting methods are expensive, time-consuming, and destructive and generally need many testing instruments. Thus, they are not suitable for use when there is an urgent need to estimate the vigor of seeds [10]. In order to achieve accurate monitoring and quality control, reliable and nondestructive testing methods are needed. Recently, nondestructive methods, such as X-ray diffraction [11,12], laser speckle analysis techniques [13,14], and measurement of electrical conductivity [15,16], were proposed for vigor detection. As a result of the low efficiency and complicated operation, the applications for seed detection based on these methods were limited [10]. Fortunately, recent studies showed that spectroscopy based techniques, such as near-infrared spectroscopy [17,18,19], nuclear magnetic resonance spectroscopy [20,21], photoacoustic spectroscopy [22], hyperspectral [23], multispectral [24], and Fourier transform near-infrared spectroscopy [25], have been developed and achieved successful applications. Especially, hyperspectral imaging (HSI) is a new technology that can record spectral and spatial information about the research object at the same time by integrating traditional images and spectral technology, a feature which is important for seed detection [10]. Thus, it demonstrated great potential in seed vigor evaluation compared with other point based spectroscopic techniques which cannot provide spatial information. Therefore, HSI has been sucessfully used in the seed variety identification of four different varieties of cotton seeds [26], roasting degrees of Columbian arabica green coffee beans [27], hybrid seeds (okra and loofah) [28], representative rice varieties in China [29,30], sweet corn seeds, and waxy corn seeds [31,32]. For vigor estimation, the HSI technique also has been widely used on corn seeds. For example, a shortwave infrared hyperspectral camera with a range of 1000–2500 nm was applied for the data analysis of corn seeds, and the results indicated that combining the visible and near-infrared hyperspectral imaging technique with multiplicative scatter correction (MSC), genetic algorithm (GA), and partial least squares regression (PLSR) was a feasible and reliable method for the determination of conductivity in corn seeds [33]. To detect the seed vigor of corn during storage, Feng et al. proposed to identifiy the seed vigor under eight different aging duration times by HSI, and the results for two varieties showed the feasibility and efficiency of HSI in evaluating seed vigor and seed aging degree [34]. In [35], HSI technique was developed to detect the extent to which corn viability was affected by heat treatment by a microwave process for three different varieties (yellow, white. and purple). Different spectra preprocessing methods, seed features of corn, and spectral ranges were compared as they were performed differently in the seed viability prediction. The accuracy of the partial least squares discriminant a nalysis (PLS-DA) to distinguish aged (heat treated) and normal (untreated) corn seeds was up to 95.6% [35]. To combine the spectral and image information of HSI for seed vigor prediction, a multi-channel data acquisition system was used for image and spectral measurement in [36]. Hyperspectral information for a 10 h period before the germination of four vigor level seeds was collected and convolutional neural networks were applied for vitality evaluation. By comparing different preprocessing and pattern recognition models, the convolutional neural network model had the best recognition by integrating the spectra and image information, and relatively high accuracy for the prediction of the four vigor grades was obtained [36]. Moisture content, which directly affects the storage time and seed germination rate, is also predicted by HSI in [37]. Based on hyperspectral images of two sides (embryo and endosperm sides) of each corn seed of four varieties, a PLSR prediction model was constructed for content prediction of several varieties. The performances of the models were compared for different preprocess and feature wavelength selections [37].

Most of the above method is mainly based on the classification of the qualitative analysis for corn seed vigor, such as the microwave heat treatment and the nontreated seed separation [33], seed aging degree evaluation [34], viable and nonviable discrimination [35], vitality level prediction [36], hardness, springiness, and resilience prediction [38]. The quantitative analysis mainly focused on the prediction of seed components related to vitality [37,39]. There is less research which has been conducted on the quantitative analysis of vigor for corn seeds, especially for sweet corn seeds. The accumulation of active starch is insufficient, and the sugar content is high, which results in low seed vigor, low seed emergence rate, weak growth potential at the seedling stage, and susceptibility to infection by pathogens. The methods of vigor detection mainly focus on the common corn seeds with consistent characteristics, such as [33,34], and high accuracy was obtained. However, the current methods were not feasible for sweet corn as there is a large difference among varieties after drying. In current seed vigor measurement, the seedling emergence rate in the field is mostly considered, but the ability of seedling emergence (such as root length and seedling length) has always been ignored. The ability of seedling emergence is important because it is the basis for ensuring the later growth of seeds and improving crop yield. In order to ensure the systematicity and integrity of the vitality assessment, it is particularly important to evaluate the seed vigor and emergence ability of sweet corn.

Conversely, the feature extraction of hyperspectral data is particularly important for the vigor prediction and variety classification for sweet corn. Many researchers have proposed various feature selection and evaluation methods; however, the existing methods, such as GA and successive projections algorithm (SPA), are based on the global data distribution, and the impact of each spectral band on the seed germination prediction has not been analyzed. A key feature selection method based on the correlation between vitality and each band was calculated and then used for seed germination prediction. In this paper, sweet corn seed vigor detection is studied through feature correlation among the hyperspectral data and the seed variety. Based on different feature reduction methods, the key characteristics of hyperspectral images of seeds were extracted, and three different regression models were constructed to determine the prediction of seed vigor for sweet corn. To evaluate the HSI in sweet corn seed viability, a germination test was conducted to determine seed viability as reference. The accuracy and superiority of different feature selection methods and regression algorithms are compared to determine the most effective methods for nondestructive testing of seed vigor.

## 2. Materials and Methods

### 2.1. Sample Preparation and Data Collection

In this paper, the sweet corn seeds of Lilixiangtian were sealed in plastic bags and stored in a refrigerator at 4 °C until the HSI process, and their moisture content was 7–8%. Sweet corn seeds were purchased from a seed company (FMYS Technology Ltd., Beijing, China), and 89 corn seeds with similar size and complete structure were randomly selected and numbered. The Gaiasky Mini visible/near-infrared hyperspectral imaging system (Dualix Instrument Co., Ltd., Sichuan, China), with 386.7–1016.7 nm band, including a CCD camera with a spectral resolution of 3 nm ± 0.5 nm (Sony, icx285, Tokyo, Japan) and two 50-W LED lights, was used. The Kennard–Stone method was used to divide the spectral data into the 73 training samples and 16 testing samples, and then the accuracy of model regression and germination prediction was calculated by the test set. Each seed was placed in the same arrangement (8 rows × 8 rows), with the embryo side facing up, and then scanned in 1.2 mm/s rows, with an exposure time of 15 ms, to obtain 256 spectral images.

**Figure 1:**
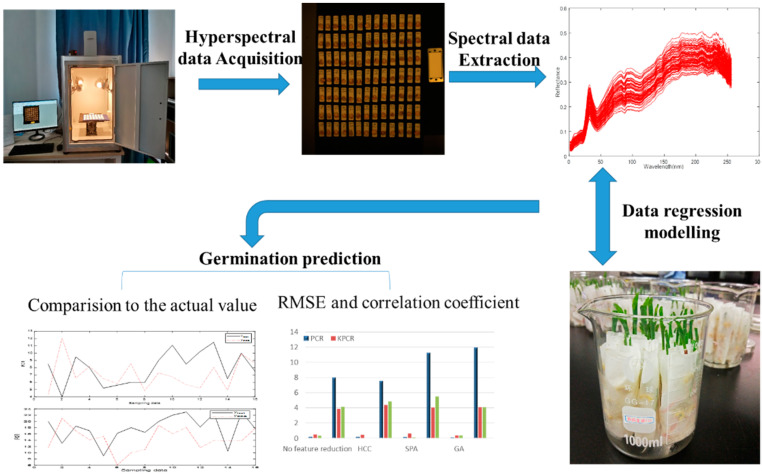
Schematic overview of the analytical procedure for the germination prediction of sweet corn seed.

The seedling length and root length after seed germination reflect the vigor of seeds. In this study, these two indexes were used to monitor the seed vigor. The seeds were soaked in water under the same conditions, and the germination conditions were observed continuously with the paper tower germination method. If the seeds germinated, the root length and the whole seedling length of the seedlings were measured seven days later based on the longest root length. A schematic overview of the analytical procedure, including the hyperspectral data collection, germination experiment, regression modeling, and germination prediction for every single seed, is given in Figure 1.

### 2.2. Extraction of the Region of Interest

The spectral data of sweet corn seeds obtained by the spectrometer contained not only useful information but also random noise. In order to reduce the noise interference caused by spectral data, 220 middle bands from 430.1 to 971.5 nm were selected for data analysis. The region of interest (ROI) is created by dividing the embryo and endosperm parts of seeds with ellipses, and then the average spectral data in ROI are extracted by ENVI 5.1 (ITT Visual Information Solutions, Boulder, CO, USA).

The white light correction data *I_white_* are obtained by scanning the standard white correction plate with 99.99% reflectance, and the dark light correction data *I_black_* are obtained by covering the lens of the device. The white and black light correction are performed on the collected image using two reference values of the original spectral image (*I_raw_*), and the corrected hyperspectral data *I* are calculated according to Equation (1) [40]:

(1) $$I=\frac{I_{raw}-I_{black}}{\;I_{white}-I_{black}}$$

### 2.3. Optimal Wavelength Selection

Each extracted spectrum consisted of 220 spectral bands ranging from 430.1 to 971.5 nm. As a small number of variables can reduce redundancy and computation, it was expected that fewer bands that represent most of the useful information should be obtained. To obtain the most relevant characteristic band of seed vigor, the highest correlation coefficient (HCC) based method was constructed between the hyperspectral data of each band and the root length of seeds or seeding length. The procedure of determining the HCC consists of correlation coefficient calculation and key feature selection. First, the correlation coefficient between each hyperspectral band and the germination results (root length of seedlings, root length) was calculated. Second, the correlation coefficient of each hyperspectral band and the germination feature were established, and the characteristic band with the highest correlation was selected. Finally, the regression model between the key characteristic band of seed and the root length (seedling length) was established, as shown in Formula (2):

(2) $$\rho_{\mathrm{ij}}=\frac{x_{i}y_{j}}{\sqrt{var(x_{i})var(y_{j})}}$$

where *ρ*_ij_ is the correlation coefficient between the *i*th-band data ***x***_i_ and the root length/seedling length of seedlings; *var*(***x****_i_*) is the variance of the *i*th-band ***x****_i_* of the hyperspectral data for seeds; *var*(***y****_i_*) is the variance of the root length/seed length corresponding to the seeds after germination. The correlation coefficients were sorted, and the band with a strong correlation was selected.

Two methods, the successive projection algorithm (SPA) and genetic algorithm (GA), were used to select the optimal wavelengths for the hyperspectral data. In the GA, the seedling root length/seedling length was set as the fitness function, and the hyperspectral data feature for seeds was set as the genetic gene to randomly generate the primary generation population. The maximum number of iterations was set as 100, and the feature variable corresponding to the highest fitness function was selected to realize the optimal band for sweet corn seed [41].

### 2.4. Prediction Model Construction for Seed Germination

In this paper, principal component regression (PCR), partial least squares (PLS), and kernel principal component regression (KPCR) were used to establish the regression relationship between the hyperspectral data of seeds and the seedling length/root length after germination. The hyperspectral method provides a comprehensive and complete state for the external morphology and internal structure of seeds. Through this type of regression model, the relationship between the hyperspectral data of seeds and their corresponding seedling length (root length) that characterize the seeds’ germination was obtained, which provides a theoretical basis for the nondestructive testing of seed vigor.

**Figure 2:**
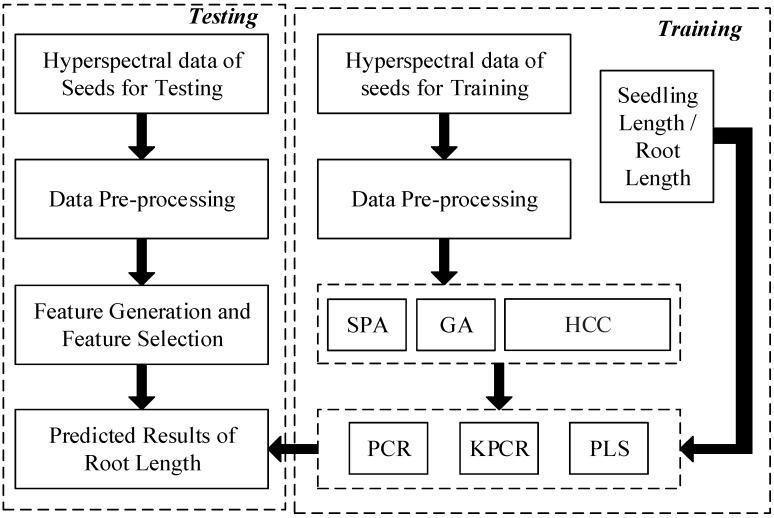
The detailed procedure of the training and testing model for germination prediction of sweet corn seeds. (SPA represents the successive projections algorithm. GA represents the genetic algorithm. HCC represents the highest correlation coefficient. PCR represents the principal component regression algorithm. KPCR represents the kernel principal component regression algorithm. PLS represents the partial least squares algorithm).

The training data of corn seeds were collected as $X\left(x_{1},\ldots ,x_{2}\right)\in R^{D\times n}$ (*D* is 256 wavelengths of hyperspectral spectrum). A flow chart of the model construction and germination prediction is shown in Figure 2.

### 2.5. Regression Algorithm

PCR is a multiple regression analysis method; it aims to solve the problem of multicollinearity among independent variables for data regression. In PCR, the collected training data were reduced into the low-dimensional space by PCA, and then a linear regression, which represents the data relation between the original data and the output variables, was constructed between the projections and output variables. KPCR is a nonlinear extension of the linear regression algorithm PCR, which maps the high dimension data through the nonlinear function, and then the regression relationship of training samples through PCR is obtained. The kernel function selected by KPCR is the Gaussian radial basis function, that is $k\left(x,y\right)=\exp(-{∥x-y∥}^{2}/\sigma )$, where σ is selected as σ=50 [42]. In this study, the three methods were applied for the data regression between the hyperspectral data of the seed and their root length/the seedling length.

## 3. Results and Discussion

**Figure 3:**
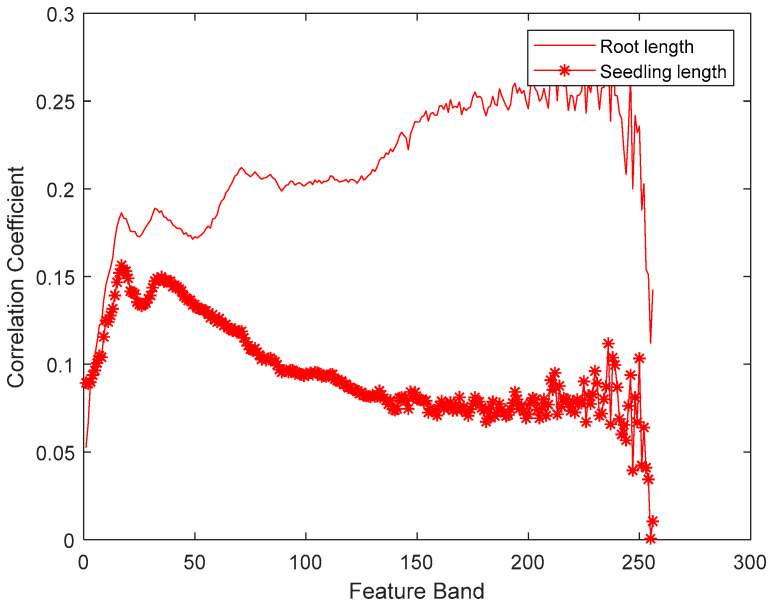
The correlation coefficient between the hyperspectral data with the seedling length and root length.

As a large number of seed spectral data variables were collected, there was collinearity, redundancy, and even noise and interference among many spectral variables, which can lead to long calculation time in the modeling of spectral data regression. In this experiment, three methods of correlation coefficient selection, GA, and SPA were used to select the characteristic band of spectral data, and the characteristic variables were used to establish the regression model. Seedling length and seedling root length are two effective indexes to reveal the seed vigor [34]. In this paper, the relevant characteristic band associated with seed vigor was selected based on the HCC. Figure 3 shows the correlation coefficient of each feature band with seedling length and seedling root length. It can be seen from Figure 3 that the correlation between hyperspectral data and seedling root length was stronger than that with seedling length. More importantly, the correlation coefficients of different band data with seedling length and seedling root length were different. We can notice from Figure 3 that the seedling length is strongly related to the data in the low frequency band, and the root length is correlated with the data in the medium-high frequency band. In this paper, the first 100 feature bands which have high correlation coefficients as computed by Equation (2) were selected for the seedling length and root length prediction model.

The model based on seed hyper spectral data and root length/seedling length of the seeds was constructed by KPCR, PCR, and PLS. The performance of HCC, SPA, and GA for feature selection was also compared with the results without feature reduction. Table 1 and Table 2 show the regression and prediction results for root length and seedling length based on different feature selection and regression methods, where the RMSE and correlation coefficient (CC) criterion are given. The larger value of the correlation coefficient and lower RMSE indicates the high prediction accuracy and correlation between the actual value and the predicted value.

From the comparison results in Table 1, the root length prediction based on HCC+KPCR gives superior predictions to the other models with regard to both evaluation criteria. It should be noted that the model regression of the KPCR method is consistent with the actual output trajectory, with minimal deviation. The kernel extension model performs better than the linear models PCR and PLS, which indicates that it can improve the nonlinear regression performance by taking kernel trick into consideration, as data relations in seeds are generally nonlinear. Similar to the classification results in classifying seeds, the nonlinear models, such as KPCR, showed better performance than the linear model for the hyperspectral based method [28,43]. Among the feature selection methods, the method based on the data correlation proposed in this study obtained the highest data correlation for all three regression models. Similar to the proposed method, the bands with low correlation were omitted from the classification of hyperspectral data in [43]. The proposed paper further proves the effectiveness of the hyperspectral data modeling by considering the spectral correlation in band selection. From Table 1, we can notice that the best correlation coefficient was 0.7805, which indicated the relevance between the hyperspectral data and root length of seeds. The result further verified the feasibility of the hyperspectral method in seed germination prediction, as in many other studies [33,34,35,36,37].

The prediction results of the whole seedling length for the seeds after germination are shown in Table 2. It can be seen from Table 2 that the highest correlation coefficient is 0.6 based on SPA+KPCR, and this is consistent with the results of the KPCR algorithm in Table 1. The results indicated that the correlation between the hyperspectral data of corn and the whole seedling length is small, so it is difficult to accurately predict the whole seedling length through hyperspectral images. The correlation between hyperspectral characteristics and different germination characteristics (seedling root length, seedling length,) was different, which was consistent with the results in [44]. The strong correlation between the seed and the root length after germination was also verified by the experiment in [45]. HCC did not provide the optimal results because the data correlation between hyperspectral and seedling length is poor, which is also shown in Figure 3. In other words, they have complex nonlinear correlations. HCC is the method used for feature selection based on the data correlation between each single band, so it is difficult to find the feature band under complex nonlinear correlation for all the hyperspectral data.

**Figure 4:**
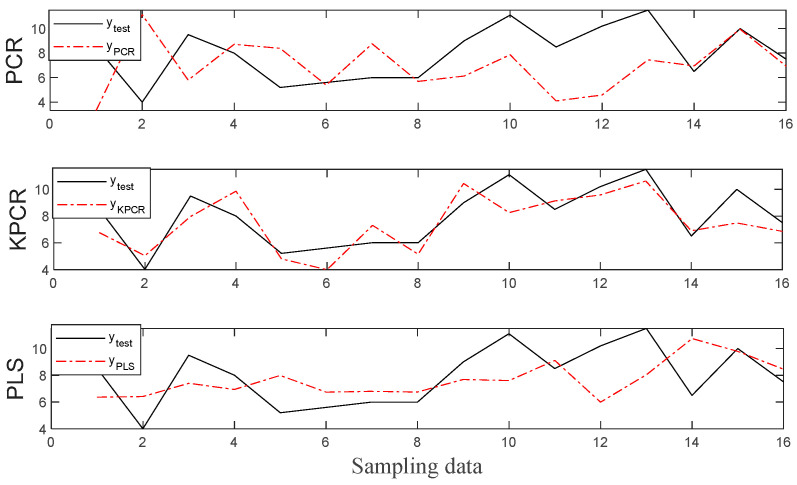
The regression results for the root length.

**Figure 5:**
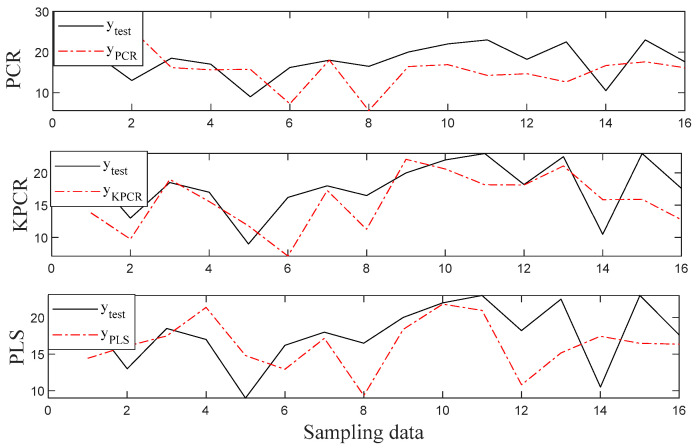
The regression results for the seedling length.

To investigate the prediction performance of the three methods for the HCC based feature selection clearly, plots of prediction results for PCR, KPCR, and PLS are shown in Figure 4 and Figure 5. It can be noticed from the figures that all the methods are capable of capturing the data variations of both root length and seedling length for the seeds because the predicted data fit the actual value for most samples. Especially, the predictions by the KPCR model coincide better with the trajectory of the actual value with the smallest deviations, while the values predicted by other methods had significant offset from the actual value.

It can be concluded from the regression results for sweet corn seeds that there is a relatively strong correlation between the hyperspectral data of seeds and their root length after germination. The effectiveness of hyperspectral data in vigor prediction, high-quality seed identification, and superior breeding for seed varieties was verified. At the same time, the validity of band selection by the data correlation was verified. KPCR is a kernel based nonlinear regression method, the good result obtained by which indicated that the relations between the hyperspectral data of seeds and the responding germination are nonlinear. Thus, the nonlinear regression model is a suitable model for the vigor detection of corn seed.

## 4. Conclusions

The vigor of sweet corn seed was predicted from hyperspectral data based on PCR, KPCR, and PLS models. The main conclusions were as follows.

(1) The accuracy of the KPCR model was higher than that of the PCR and PLS models based on the degree of correlation, and the classification accuracy of the KPCR model was 0.7805, which was much more satisfactory than the results of KPCR and PLS models (with accuracies of 0.142–0.725).
(2) The accuracy of the PCR, KPCR, and PLS models was improved by feature selection for the prediction of seedling root length for sweet corn seed, and the best prediction result was obtained after the feature selection by the correlation of hyperspectral band and the performance of germination.
(3) All of the model predictions for the root length data were more accurate than the seedling length for the test data. The above conclusions showed that the HSI technology could be improved using the KPCR model and feature selection, which can be readily applied to the prediction of root length. Further studies are needed to conduct more research on how to improve the robustness and universality of these prediction models using more varieties or samples, comparing the impacts of different HSI systems, and constructing some standard discrimination models.

In this paper, although the proposed hyperspectral based method can achieve seed vigor prediction to some extent, there are still several limitations. Firstly, only two germination characteristics for one variety of sweet corn were tested; thus, further research on the correlation among the germination characteristics and the responding hyperspectral features of different varieties are needed. Secondly, only the near-infrared (NIR) hyperspectral imaging technique was used, while the prediction of seed vigor based on the hyperspectral images in the range of 1000 to 2500 nm needs further study. Thus, further studies should be dedicated to verifying and developing feasible vigor testing methods by combing the infrared hyperspectral data for handling various seed varieties.

## Figures and Tables

**Table 1:** Comparison of hyperspectral data and root length prediction for corn seeds.

	No Feature Reduction ^1^		HCC ^2^		SPA ^3^		GA ^4^	
	CC	RMSE	CC	RMSE	CC	RMSE	CC	RMSE
PCR ^5^	0.1428	3.8086	0.4568	2.4256	0.1414	4.2311	0.0404	4.9420
KPCR ^6^	0.7199	1.6050	0.7805	1.5030	0.7252	1.6170	0.7062	1.6110
PLS ^7^	0.1446	2.8767	0.6517	2.3267	0.6873	2.0605	0.2606	3.4866

^1^ No feature reduction means that all the data features were used. ^2^ HCC represents the highest correlation coefficient. ^3^ SPA represents the successive projections algorithm. ^4^ GA represents the genetic algorithm. ^5^ PCR represents the principal component regression algorithm. ^6^ KPCR represents the kernel principal component regression algorithm. ^7^ PLS represents the partial least squares algorithm.

**Table 2:** Comparison of prediction effect between hyperspectral data and seedling length for corn seeds.

	No Feature Reduction		HCC		SPA		GA	
	CC	RMSE	CC	RMSE	CC	RMSE	CC	RMSE
PCR	0.2261	8.0484	0.2435	7.6013	0.1689	11.259	0.1280	11.9637
KPCR	0.5071	3.8987	0.4384	4.3668	0.6074	4.0527	0.4014	4.1218
PLS	0.3347	4.1678	0.0257	4.8113	0.0876	5.5121	0.3699	4.0979
